# Eggs Good and Bad

**Published:** 1870-04

**Authors:** 


### EGGS GOOD AND BAD.

THE old woman said that a good egg would either sink or swim, with its big or little end up or down, she didn't remember which, but she was certain it was one or the other. A correspondent writes that we gave the wrong directions ; but as we don't make them nor eat them, it could not be expected that we should be learned in the matter, so we give his assertion, and let the reader find out who is nearest right. He says, " Newly laid hen's eggs invariably sink and lie on the side ; cannot say as to alligator's or New York City eggs." Now that proves we are right, for we never bought an egg in New York that did not either sink or swim — those that have nothing in them swim, those that contain chickens sink.

